# Explainable and Trustworthy Artificial Intelligence in Cardiology: A Narrative Review of Clinical Applications, Operational Integration, and Future Directions

**DOI:** 10.3390/jcm15134885

**Published:** 2026-06-23

**Authors:** Mateusz Lucki, Ewa Lucka, Jacek Żak, Przemysław Mitkowski, Maciej Lesiak

**Affiliations:** 1 Department of Cardiology, Chair of Cardiology, Poznan University of Medical Sciences, 61-701 Poznan, Poland; 2 Clinical Rehabilitation Laboratory, Department of Rehabilitation and Physiotherapy, University of Medical Sciences, 60-545 Poznan, Poland; 3 Department of Logistics, Faculty of Engineering Management, Poznan University of Technology, 60-965 Poznan, Poland

**Keywords:** artificial intelligence, cardiology, explainable artificial intelligence, machine learning, deep learning, digital health, cardiovascular imaging, electrocardiography, trustworthy AI

## Abstract

**Background/Objectives:** Artificial intelligence (AI) is increasingly transforming cardiology through advanced analytical tools capable of identifying complex patterns across cardiovascular imaging, electrophysiology, and clinical datasets. Machine learning (ML) and deep learning (DL) algorithms are being integrated into echocardiography, cardiac computed tomography (CT), cardiac magnetic resonance imaging (MRI), and electrocardiography (ECG), enabling earlier diagnosis and more personalized cardiovascular care. This narrative review summarizes current clinical and organizational applications of AI in cardiology and discusses emerging concepts related to explainable and trustworthy AI. **Methods:** A narrative review was conducted according to SANRA recommendations using the PubMed, MEDLINE, Web of Science, and Scopus databases, including peer-reviewed publications from 2015 to 2026 addressing clinical, organizational, and ethical applications of AI in cardiology, with particular emphasis on cardiovascular imaging, electrocardiography, heart failure, digital health, and explainable AI frameworks. **Results:** Substantial evidence demonstrates that AI-based tools can achieve expert-level performance in cardiovascular imaging interpretation, automated electrocardiographic analysis, and clinical risk prediction. Across multiple cardiovascular settings, AI has been associated with improved diagnostic accuracy, enhanced workflow efficiency, and earlier detection of cardiovascular disease. Predictive models support risk stratification in heart failure and ischemic heart disease, while chatbots and digital health platforms may facilitate patient engagement, remote monitoring, and continuity of care. Despite these advances, important challenges remain, including algorithmic bias, limited transparency, insufficient external validation, data heterogeneity, and barriers to routine clinical implementation. Emerging explainable AI approaches may improve model interpretability, clinician confidence, and the safe adoption of AI-driven decision support systems. **Conclusions:** Artificial intelligence is rapidly evolving from a research-oriented technology into a clinically relevant component of cardiovascular care. Current evidence indicates that AI can enhance diagnostic performance, improve risk prediction, streamline clinical workflows, and facilitate more personalized management across multiple cardiovascular domains. However, the successful translation of AI into routine practice will depend on robust external validation, transparent decision-making mechanisms, regulatory oversight, and clinician acceptance. The development of explainable and trustworthy AI frameworks represents a critical step toward the safe, ethical, and sustainable integration of AI into modern cardiology.

## 1. Introduction

Cardiology has historically been one of the most technologically advanced fields in medicine, integrating imaging, hemodynamic monitoring, and electrophysiological data to guide diagnosis and therapy. The recent proliferation of large-scale, high-dimensional datasets—collectively known as *big data*—has created unprecedented opportunities for the application of artificial intelligence (AI) in cardiovascular care [1,2,3]. AI encompasses computational techniques that enable systems to simulate human cognitive functions, including perception, learning, and reasoning. Within AI, machine learning (ML) and deep learning (DL) are the most transformative subsets, allowing algorithms to autonomously detect patterns, classify disease states, and predict outcomes from complex medical data [4,5,6].

Machine learning encompasses a range of supervised, unsupervised, and reinforcement learning approaches. Supervised learning algorithms are commonly applied to disease classification, outcome prediction, and risk stratification, whereas unsupervised learning facilitates patient phenotyping and the identification of previously unrecognized disease subgroups. Deep learning, a specialized branch of ML based on multilayer artificial neural networks, has demonstrated exceptional performance in the analysis of high-dimensional cardiovascular data, including echocardiographic images, cardiac CT, cardiac MRI, electrocardiograms, and wearable sensor recordings. In particular, convolutional neural networks (CNNs) have enabled automated image interpretation, while transformer-based architectures and large language models are increasingly being explored for multimodal data integration and clinical decision support [1,2,7,8].

In cardiology, AI algorithms have been validated in nearly all major domains: from automated echocardiographic analysis and CT-derived fractional flow reserve estimation to ECG-based detection of arrhythmias and early-stage heart failure [9,10,11,12,13,14]. Beyond clinical care, AI applications now extend to hospital administration, optimizing workflow efficiency, predicting readmissions, and enhancing patient interaction through chatbots and digital assistants [15,16,17]. However, enthusiasm for AI integration must be balanced by awareness of its limitations. Challenges include data heterogeneity, potential algorithmic bias, limited interpretability (“black box” effect), and regulatory uncertainty [18,19,20]. These issues underscore the necessity for rigorous validation, transparent reporting, and clinician oversight in AI deployment.

Recognizing both the opportunities and challenges associated with AI, several international organizations have recently published guidance documents and position statements addressing its responsible implementation in healthcare. The European Association of Cardiovascular Imaging (EACVI), together with the European Association of Nuclear Medicine (EANM), has emphasized the importance of robust validation, data governance, transparency, fairness, and continuous performance monitoring of AI systems used in cardiovascular imaging [21]. Similarly, the American Heart Association (AHA) has highlighted the need for explainable and clinically trustworthy AI capable of supporting, rather than replacing, physician decision-making [22,23]. International guidance documents further emphasize that the successful implementation of AI in healthcare requires transparency, accountability, patient safety, mitigation of algorithmic bias, protection of health data, and meaningful human oversight throughout the AI lifecycle [24]. Recent guidance documents consistently emphasize that AI systems should be explainable, externally validated, continuously monitored after deployment, and implemented under appropriate human oversight to ensure patient safety and clinical reliability [21,22,23,24]. Collectively, these initiatives reflect a growing consensus that the successful integration of AI into cardiology depends not only on technical performance but also on ethical robustness, interpretability, regulatory compliance, and clinician trust.

Therefore, the purpose of this narrative review is to provide a structured overview of current AI applications in cardiology—both clinical and organizational—while discussing ethical considerations, educational implications, implementation challenges, and future directions toward explainable and trustworthy cardiovascular AI.

## 2. Review Methodology

This review was designed as a narrative review and prepared in accordance with the Scale for the Assessment of Narrative Review Articles (SANRA) recommendations [25]. The aim of this review was to provide a structured and critical overview of current and emerging applications of artificial intelligence in cardiology, with particular emphasis on clinical implementation, healthcare delivery, ethical considerations, governance, and future perspectives.

A literature search was conducted in PubMed/MEDLINE, Web of Science, Scopus, and DOAJ to identify relevant publications published between January 2015 and 31 May 2026. The search strategy combined free-text terms and controlled vocabulary related to artificial intelligence and cardiovascular medicine, including “artificial intelligence”, “machine learning”, “deep learning”, “large language models”, “chatbots”, “electrocardiography”, “echocardiography”, “heart failure”, “coronary artery disease”, “cardiac imaging”, “electrophysiology”, “interventional cardiology”, and “cardiovascular imaging”. Additional publications were identified through citation tracking of key articles, scientific statements, consensus documents, position papers, and clinical practice guidelines issued by major professional societies, including the European Society of Cardiology (ESC), American Heart Association (AHA), American College of Cardiology (ACC), and the European Association of Cardiovascular Imaging (EACVI).

Because this work follows a narrative review methodology, no formal systematic-review procedures, quantitative meta-analysis, or study-quality scoring system were applied. Instead, publications were selected based on their scientific relevance, methodological rigor, clinical impact, and contribution to the understanding of contemporary AI applications in cardiovascular medicine. Particular emphasis was placed on landmark original investigations, multicenter validation studies, systematic reviews, consensus statements, position papers, and recent publications addressing implementation, explainability, governance, safety, regulatory considerations, and ethical aspects of AI.

The final reference list comprised 98 citations. Of these, 97 references were included to support the narrative synthesis presented in this review, whereas one reference corresponded to the SANRA methodological framework used to guide the preparation and reporting of the manuscript [25]. The selected references were chosen to provide a balanced overview of the rapidly evolving field of artificial intelligence in cardiology, encompassing foundational studies, clinical validation cohorts, professional society recommendations, regulatory and ethical frameworks, and recent developments in cardiovascular AI.

The evidence synthesized in this review was organized into two major domains: (1) clinical applications of AI in cardiology, including cardiovascular imaging, electrocardiography, heart failure, coronary artery disease, electrophysiology, and interventional cardiology; and (2) healthcare system applications, including workflow optimization, patient engagement through conversational AI, research integration, regulatory frameworks, and the responsible implementation of AI technologies in clinical practice.

Reference [25] represents the SANRA methodological guidance used for the preparation and reporting of this narrative review and was included solely for methodological purposes rather than as part of the reviewed evidence on artificial intelligence in cardiovascular medicine.

**Figure 1:**
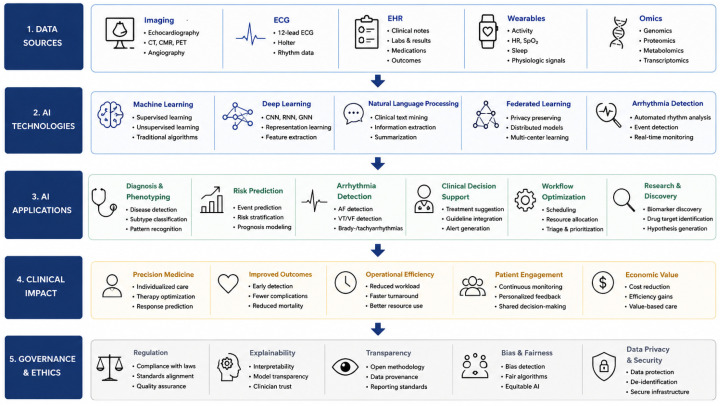
AI ecosystem in cardiology.

**Figure 2:**
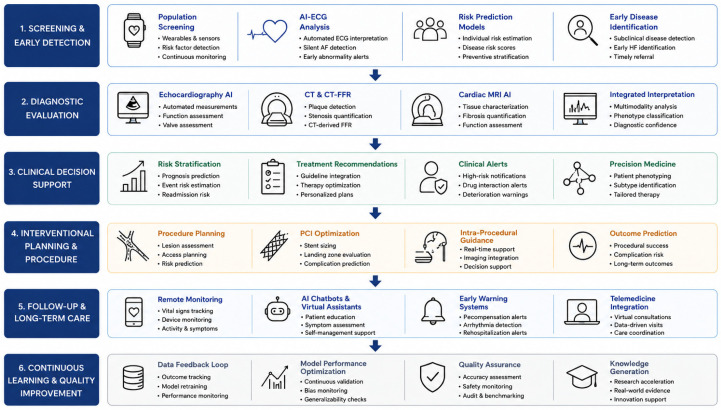
Application of Artificial Intelligence Across the Cardiovascular Care Continuum.

Figure 1 provides a conceptual overview of the AI ecosystem in cardiology, illustrating the interplay between key data sources, core AI methodologies (ML, DL, NLP), and the clinical and organizational domains in which these tools are applied. The framework also incorporates essential ethical and governance foundations, including privacy protection, fairness, explainability, and regulatory oversight. By outlining how data acquisition, computational methods, and healthcare workflows converge, Figure 2 serves as a visual guide for understanding the structure and logic of the subsequent sections of this review.

## 3. Clinical Applications of AI in Cardiology

### 3.1. Echocardiography and Multimodal Cardiovascular Imaging

AI has dramatically reshaped cardiovascular imaging workflows by automating repetitive tasks such as image acquisition, segmentation, and interpretation [13,21,22]. In echocardiography, deep learning (DL) models based on convolutional neural networks (CNNs) achieve highly accurate classification of cardiac views, chamber delineation, and quantification of left ventricular ejection fraction (LVEF) [10,14]. Ouyang et al. demonstrated that a video-based DL model could estimate LVEF with expert-level accuracy, outperforming manual measurements in reproducibility and speed [5].

In cardiac computed tomography angiography (CCTA), machine learning enables automated coronary calcium scoring, detection of non-calcified plaque components, and evaluation of high-risk features such as positive remodeling and the napkin-ring sign [12,15,18]. Similarly, in cardiac magnetic resonance (CMR) imaging, DL algorithms facilitate myocardial tissue characterization, quantifying fibrosis and perfusion abnormalities [13,20,21].

Furthermore, AI-derived fractional flow reserve (CT-FFR) integrates anatomical and functional data, improving the diagnostic accuracy for ischemia compared with conventional CCTA alone [17,19]. In combination, multimodal AI systems integrating echo, CMR, and CT data promise precision phenotyping of cardiac disease, reducing interobserver variability and enhancing diagnostic reproducibility [13,21,22].

Beyond common cardiovascular conditions, AI-assisted multimodality imaging may also improve the assessment of less frequently studied valvular diseases. For example, advanced machine learning algorithms integrating echocardiographic, cardiac magnetic resonance, and cardiac computed tomography data may facilitate more accurate quantification of pulmonary stenosis and pulmonary regurgitation, improve longitudinal follow-up, enable earlier detection of right ventricular remodeling, and support optimal timing of intervention in patients with congenital heart disease [26].

### 3.2. Electrocardiography and Arrhythmia Detection

AI-driven electrocardiographic analysis has emerged as one of the most successful implementations of ML in clinical cardiology [6,8,11]. Supervised neural networks trained on large ECG databases can detect subtle, subclinical signatures of disease invisible to the human eye. Attia et al. demonstrated that an AI-enabled ECG could identify asymptomatic left ventricular dysfunction, offering a scalable and low-cost screening tool for early heart failure [8]. Similarly, AI models can accurately detect atrial fibrillation, ventricular tachycardia, prolonged QT, electrolyte disturbances, and ischemia [6,27,28]. The Apple Heart Study showed that smartwatch-based photoplethysmography (PPG) combined with AI analysis enables large-scale atrial fibrillation screening and integration into telemedicine pathways [29]. Other studies highlight that AI-ECG can infer parameters such as age, sex, and serum potassium levels, illustrating its diagnostic potential beyond rhythm interpretation [11,28]. Integration of AI-based ECG tools into remote monitoring systems facilitates continuous rhythm surveillance and early clinical intervention, reducing unplanned admissions for arrhythmia-related complications [30].

Recent evidence further highlights the expanding role of AI in cardiac electrophysiology. Beyond arrhythmia detection, AI-based approaches are increasingly being applied to risk stratification, prediction of atrial fibrillation recurrence, sudden cardiac death assessment, optimization of catheter ablation strategies, and personalized management of complex rhythm disorders. These developments underscore the growing integration of AI into contemporary electrophysiology and support its potential to enhance precision-guided arrhythmia care [31].

A practical clinical example is the use of AI-enabled ECG algorithms in primary care and emergency departments to identify previously unrecognized left ventricular systolic dysfunction in patients presenting with dyspnea, allowing earlier referral for echocardiography and initiation of guideline-directed therapy [8,9]. In addition, smartwatch-based AI systems may detect silent atrial fibrillation in asymptomatic individuals, enabling timely anticoagulation and stroke prevention [32].

### 3.3. Heart Failure: Prediction, Phenotyping, and Therapy Optimization

Heart failure (HF) is a clinical domain where AI shows exceptional promise. Predictive models using electronic health records (EHRs), biomarkers, and imaging data can forecast hospitalization or mortality up to 30 days before clinical decompensation [26,33,34].

Machine learning enhances risk stratification by identifying nonlinear relationships between clinical variables that traditional models may overlook [33,35]. Unsupervised learning approaches have identified novel HF phenotypes that go beyond the classical ejection fraction classification. For instance, ML-based clustering of patients with HFpEF has uncovered subgroups differing in comorbidity burden, cardiac geometry, and outcomes [26,33]. Such AI-based phenotyping enables personalized therapy selection, particularly for optimizing guideline-directed medical therapy (GDMT) involving SGLT2 inhibitors, ARNI, and mineralocorticoid receptor antagonists [27,33]. Moreover, DL-based decision-support systems are now being developed to recommend drug titration sequences and follow-up frequency, enhancing adherence to evidence-based management [26,28].

In clinical practice, AI-based phenotyping may help identify patients with HFpEF who are more likely to benefit from specific therapeutic strategies, while predictive models can alert clinicians to impending decompensation and support proactive adjustment of treatment before hospitalization becomes necessary [26,27,33].

### 3.4. Coronary Artery Disease and Interventional Cardiology

In the realm of coronary artery disease (CAD), AI supports both diagnostic and interventional decision-making. Machine learning applied to CCTA improves the detection of hemodynamically significant stenoses and characterizes plaque morphology [12,15,18,36]. CT-based fractional flow reserve estimation (CT-FFR) provides a non-invasive assessment of ischemia, guiding the need for revascularization with high diagnostic performance [17,19]. In the catheterization laboratory, real-time AI-assisted systems analyze angiographic sequences, calculate vessel dimensions, and assist in stent sizing and placement [37,38]. Robotic-assisted PCI platforms with embedded AI modules are being developed to standardize procedures, reduce radiation exposure, and improve precision [39]. Furthermore, AI models are being used to predict periprocedural complications, optimize contrast media use, and personalize antithrombotic strategies based on individual risk profiles [34,40]. Collectively, these advances illustrate the profound integration of AI into the full continuum of CAD management—from diagnosis to intervention.

A practical clinical example is the use of AI-assisted quantitative coronary angiography during percutaneous coronary intervention, where automated lesion assessment and vessel sizing may improve procedural planning and optimize stent selection. In patients with intermediate coronary stenoses, AI-enhanced CT-FFR analysis may support decision-making regarding revascularization and help avoid unnecessary invasive procedures. Emerging AI-guided platforms have also demonstrated the potential to enhance procedural precision and reduce operator variability during complex coronary interventions [37,41].

### 3.5. Integration of Artificial Intelligence Across the Cardiovascular Care Pathway

Beyond individual diagnostic and therapeutic applications, artificial intelligence is increasingly being integrated across the entire cardiovascular care continuum (Figure 2). AI-supported technologies contribute at multiple stages of patient management, from population screening and diagnostic evaluation to treatment optimization, remote monitoring, and continuous learning healthcare systems. These approaches include wearable devices, AI-enhanced electrocardiography, and predictive risk models [29,42,43,44]. Such approaches facilitate the identification of individuals at increased risk of cardiovascular disease, heart failure, or atrial fibrillation before overt clinical manifestations become apparent.

For example, AI-supported wearable devices may continuously analyze physiological signals and automatically alert healthcare providers to early signs of atrial fibrillation, heart failure deterioration, or abnormal heart rhythms, enabling earlier intervention and reducing the need for emergency hospitalization [29,42,43,45,46,47,48,49,50].

During diagnostic evaluation, AI enhances the interpretation of multimodality cardiovascular imaging, including echocardiography, coronary computed tomography angiography, and cardiac magnetic resonance imaging [13,21,22,51]. These tools improve efficiency, reduce interobserver variability, and enable more precise disease phenotyping. Integration of imaging, laboratory, electrocardiographic, and electronic health record data further supports risk stratification, treatment selection, and implementation of guideline-directed medical therapy [22,23,26,28,32]. In interventional cardiology, AI-assisted systems may optimize procedural planning, lesion assessment, stent sizing, and prediction of procedural outcomes [37,39,52,53].

The role of AI extends beyond acute clinical decision-making into longitudinal patient management. Remote monitoring platforms, conversational agents, and telemedicine solutions facilitate symptom surveillance, medication adherence, and early identification of clinical deterioration [30,42,45,46,47,48,49,50]. At the same time, continuous-learning frameworks based on real-world data support ongoing model refinement, quality improvement, and the development of learning healthcare systems [54,55,56,57,58,59].

A practical example of pathway-wide AI integration is the management of patients with chronic heart failure using remote monitoring ecosystems. Data from wearable sensors, home blood pressure measurements, implanted cardiac devices, electronic health records, and AI-based predictive models may be continuously analyzed to identify early signs of clinical deterioration. Such systems can automatically alert healthcare teams, facilitate timely therapeutic adjustments, and potentially reduce emergency department visits and hospital readmissions [30,42,45,46,47,48,49,50]. Taken together, these developments highlight the evolution of AI from isolated diagnostic tools toward an integrated clinical support ecosystem capable of enhancing personalized, efficient, and data-driven cardiovascular care throughout the entire patient journey [33,44,53,59].

## 4. Organizational and Administrative Applications of AI in Cardiology

### 4.1. Workflow Optimization and Predictive Operations

The integration of AI into hospital operations represents a paradigm shift from reactive to predictive and adaptive healthcare management [60,61,62]. Cardiology departments, in particular, face significant logistical challenges, including unpredictable patient inflows, limited intensive care capacity, and high procedural demand in catheterization laboratories. AI-driven forecasting systems analyze historical and real-time data to anticipate patient admissions, optimize staff allocation, and prevent bottlenecks in clinical workflows [63,64]. Predictive models based on ML can estimate bed occupancy rates, emergency department congestion, and even equipment utilization patterns, allowing administrators to preemptively adjust resource distribution [63,64].

For example, integrating AI into EHR and hospital management software enables early identification of high-risk patients requiring escalation of care, reducing unplanned readmissions and length of stay [62,64,65]. Furthermore, natural language processing (NLP) tools automate data extraction from unstructured clinical notes, creating real-time quality dashboards for metrics such as door-to-balloon time in myocardial infarction or adherence to GDMT in heart failure [55,65]. Federated learning platforms allow the development of predictive models across multiple institutions without transferring patient data, maintaining compliance with privacy regulations while improving generalizability [56,66].

### 4.2. Chatbots and Conversational Agents in Cardiology

AI-powered chatbots have emerged as valuable tools for enhancing patient engagement, education, and adherence [45,46,47]. Based on natural language processing, these conversational agents interact with patients through mobile or web interfaces, providing education about medications, lifestyle modifications, and warning symptoms [45,46,47]. In cardiology, chatbots have been successfully deployed in heart failure monitoring, cardiac rehabilitation, and post-PCI follow-up [46,48]. They assist in collecting self-reported parameters (e.g., body weight, heart rate, blood pressure) and can trigger alerts for clinicians when concerning trends are detected. These systems improve patient satisfaction by offering continuous support while reducing the administrative burden on clinical staff [48,49]. Recent advances in generative AI (such as large language models, LLMs) have enabled chatbots to engage in more natural, context-aware communication with patients. When integrated with EHR systems and safety filters, they can facilitate triage, appointment scheduling, and post-discharge education [49,67]. However, accuracy, bias mitigation, and strict clinical oversight are crucial, particularly when chatbots provide condition-specific recommendations [50,68]. Professional societies such as the ESC emphasize the need for human-in-the-loop validation and explainable reasoning in all conversational AI systems deployed in healthcare [23,67].

Despite their considerable potential, large language model (LLM)-based chatbots remain associated with important limitations and safety concerns. Hallucinations may result in the generation of clinically inaccurate information, while excessive reliance on automated responses may provide inappropriate reassurance to patients experiencing potentially serious cardiovascular symptoms.

Additional challenges include algorithmic bias, privacy concerns, cybersecurity vulnerabilities, prompt injection attacks, medicolegal responsibility for AI-generated recommendations, and the lack of longitudinal accountability for patient outcomes [67,68,69,70]. Consequently, conversational AI systems should operate within validated escalation pathways that ensure timely referral to qualified healthcare professionals whenever clinical uncertainty, high-risk symptoms, or potentially urgent conditions are identified. Continuous monitoring, periodic validation, explainability, and human oversight remain essential prerequisites for the safe deployment of LLM-based systems in cardiovascular care [23,67,71].

### 4.3. Research Acceleration and Data Integration

AI-driven analytics are revolutionizing cardiovascular research by automating data curation, study design, and statistical analysis. Natural language processing (NLP) and optical character recognition (OCR) systems can extract structured information from legacy datasets, imaging archives, and clinical notes, enabling rapid registry updates and cohort creation [54,56]. This has been particularly valuable in multicenter registries such as HEROES and PROMISE, where harmonization of heterogeneous data sources accelerates translational research. Federated learning enables multi-site model training without direct data sharing, ensuring compliance with GDPR and ethical standards while maintaining model accuracy [55,65]. This approach has already been validated for AI-based risk prediction in heart failure and plaque characterization in CCTA across European datasets [33,55,57]. In addition, AI facilitates automated literature synthesis, assisting researchers and clinicians in keeping pace with the exponential growth of publications. For example, ML-based bibliometric systems can identify emerging trends in cardiovascular innovation and predict citation trajectories or topic evolution within the domain [56,67]. The successful translation of AI technologies into routine cardiovascular practice also requires rigorous methodological evaluation and transparent reporting. Several dedicated frameworks have been developed to address these challenges, including SPIRIT-AI for clinical trial protocols, CONSORT-AI for reporting randomized clinical trials involving AI interventions, and DECIDE-AI for the early-stage clinical evaluation of decision-support systems [59,60,72,73]. Adoption of these standards may improve reproducibility, transparency, patient safety, and regulatory acceptance, thereby facilitating the responsible implementation of AI across cardiovascular medicine [60,72,73]. Collectively, these tools foster a learning healthcare system—a continuously adaptive ecosystem in which patient data, clinical research, and administrative processes converge to inform evidence-based decision-making in real time [56,58].

## 5. Ethical, Legal, and Educational Aspects

The implementation of artificial intelligence (AI) in cardiology raises significant ethical, legal, and educational challenges that must be addressed to ensure safe, equitable, and responsible adoption.

### 5.1. Algorithmic Bias and Data Equity

AI models are only as reliable as the data on which they are trained. If datasets underrepresent specific populations—such as women, ethnic minorities, or older adults—models may inadvertently reproduce or amplify existing healthcare disparities [69,74].

Studies have demonstrated performance gaps in AI-driven ECG interpretation and imaging segmentation across demographic subgroups [72,75]. Addressing these inequities requires balanced dataset design, transparent performance reporting, and inclusion of subgroup-specific validation metrics. The ESC’s 2024 [76] position paper on AI ethics highlights the obligation to perform algorithmic fairness auditing before clinical deployment [69].

### 5.2. Transparency and Explainability

AI systems, particularly those based on deep learning, often function as “black boxes,” making it difficult to interpret their reasoning process [76,77]. To improve clinician trust, the emerging field of Explainable AI (XAI) focuses on creating interpretable models and visualization techniques such as saliency maps, feature attribution scores, and attention-weight visualization [77,78,79]. However, post hoc explanations may not always reflect the true internal logic of an algorithm, necessitating prospective validation of interpretability methods [76,78,80].

### 5.3. Regulatory Frameworks and Legal Accountability

Regulatory oversight is rapidly evolving. In Europe, the EU Artificial Intelligence Act (2024) classifies most clinical AI systems as “high-risk,” mandating strict requirements for transparency, traceability, and human oversight [79,81,82,83,84]. This framework complements the Medical Device Regulation (MDR) and ensures that AI tools undergo continuous performance monitoring throughout their lifecycle. In the United States, the FDA’s Action Plan for AI/ML-based Software as a Medical Device (SaMD) emphasizes adaptive learning oversight and the need for post-market surveillance [84,85]. Institutions deploying AI must therefore implement AI governance structures including algorithm inventories, version control, bias monitoring, and rollback mechanisms [80,81,82,83]. These processes ensure accountability and protect patients from model drift—gradual performance deterioration as real-world data evolve over time [86].

### 5.4. Education and Clinical Integration

Finally, sustainable implementation of AI in cardiology depends on clinician education and digital literacy. Cardiologists must understand both the strengths and limitations of AI outputs, maintaining a “human-in-the-loop” approach that combines algorithmic insights with clinical reasoning [43,86]. Several medical schools and professional societies, including the ESC and AHA, have introduced structured curricula on AI ethics, data science, and informatics, underscoring the importance of interdisciplinary collaboration between clinicians, engineers, and data scientists [87,88,89,90].

### 5.5. Current Barriers and Limitations to Clinical Implementation of AI

Despite substantial advances in artificial intelligence, several important barriers continue to limit its widespread implementation in routine cardiovascular practice. One of the major challenges is the limited external validity and generalizability of many AI models. Algorithms are often developed and validated using retrospective datasets originating from specific institutions or highly selected populations, which may reduce their performance when applied to diverse real-world clinical settings [68].

Another important limitation is data quality and interoperability. Clinical data are frequently fragmented across different electronic health record systems, imaging platforms, and wearable devices, creating challenges for model development, validation, and deployment [65,91]. Missing data, inconsistent documentation, and differences in data standards may further compromise model reliability and reproducibility [52].

The “black-box” nature of many deep learning algorithms remains a significant obstacle to clinician trust and adoption. Although explainable AI techniques have improved model interpretability, many systems still provide limited insight into the rationale behind specific predictions or recommendations [76,77,91]. Consequently, physicians may be reluctant to rely on AI-generated outputs when managing complex clinical situations.

Practical implementation challenges also persist. Integration of AI tools into existing clinical workflows often requires substantial financial investment, technical infrastructure, regulatory oversight, and personnel training [6,60,80]. Healthcare institutions must additionally establish governance frameworks to continuously monitor model performance, detect algorithmic drift, and ensure compliance with evolving legal and ethical requirements [80,81,83].

Finally, prospective evidence demonstrating improvements in hard clinical outcomes remains relatively limited. While numerous studies have reported excellent diagnostic performance, fewer investigations have shown that AI-assisted decision-making translates into reduced mortality, lower hospitalization rates, improved quality of life, or cost-effectiveness in routine practice [23,43]. Addressing these limitations will be essential for achieving safe, equitable, and sustainable integration of AI into cardiovascular care.

## 6. Future Directions

The next decade will likely redefine the role of AI in cardiology through convergence of multimodal learning, explainable intelligence, and human-centered design.

### 6.1. Multimodal and Longitudinal Learning

Future AI systems will integrate diverse data modalities—genomic, proteomic, imaging, electrophysiological, and behavioral—into unified models capable of longitudinal prediction [92,93,94]. This approach, often termed digital twin cardiology, allows for virtual simulations of individual patient trajectories and personalized treatment optimization [93,95,96].

### 6.2. Explainable and Trustworthy AI

As models become more complex, explainability will remain essential. Transparent, auditable systems that quantify uncertainty and provide contextual explanations will improve clinician confidence and patient safety [73,88,92]. Trustworthy AI frameworks, incorporating bias auditing and continual learning safeguards, will enable safe scaling across healthcare systems [71,96].

### 6.3. Integration with Wearables and Chatbots

AI-powered wearables will extend cardiovascular monitoring into patients’ daily environments, capturing dynamic physiological trends that inform proactive care [41,42]. Chatbots and conversational AI will evolve from static question–answer systems to context-aware virtual companions capable of supporting self-management, medication adherence, and emotional well-being [43,48,68].

### 6.4. Interdisciplinary and Policy Collaboration

Finally, advancing AI in cardiology requires a multidisciplinary ecosystem encompassing clinicians, ethicists, regulators, and technology developers [23,96]. Global standardization of data interoperability, validation protocols, and performance benchmarks will be critical to ensure reproducibility and safety across diverse populations [52,55]. The ESC and EACVI emphasize that the ultimate goal is AI for equity, transparency, and precision, not automation for its own sake [44,77,93,94].

## 7. Conclusions

AI has transitioned from experimental innovation to an operational component of modern cardiology. From automated image interpretation and ECG-based risk detection to predictive hospital management and patient engagement via chatbots, AI now spans both the clinical and organizational dimensions of cardiovascular care. While these technologies offer unprecedented opportunities to improve efficiency and outcomes, their deployment must be guided by rigorous validation, ethical governance, and continuous education. AI should not replace clinical expertise but rather enhance human decision-making, fostering a new era of precision, empathy, and data-driven cardiovascular medicine.

## Data Availability

No new data were created or analyzed in this study. Data sharing is not applicable to this article.

## Abbreviations

AI	Artificial Intelligence
CCTA	Coronary Computed Tomography Angiography
CMR	Cardiac Magnetic Resonance
CNN	Convolutional Neural Network
CT-FFR	Computed Tomography-Derived Fractional Flow Reserve
DL	Deep Learning
ECG	Electrocardiography
GDMT	Guideline-Directed Medical Therapy
HF	Heart Failure
LLM	Large Language Model
ML	Machine Learning
NLP	Natural Language Processing
